# Drug Repurposing and Lysosomal Storage Disorders: A Trick to Treat

**DOI:** 10.3390/genes15030290

**Published:** 2024-02-25

**Authors:** Bruno Hay Mele, Federica Rossetti, Maria Vittoria Cubellis, Maria Monticelli, Giuseppina Andreotti

**Affiliations:** 1 Department of Biology, University of Napoli “Federico II”, Complesso Universitario Monte Sant’Angelo, Via Cinthia, 80126 Napoli, Italy; bruno.haymele@unina.it (B.H.M.); fed.rossetti@studenti.unina.it (F.R.); cubellis@unina.it (M.V.C.); 2 Institute of Biomolecular Chemistry ICB, CNR, Via Campi Flegrei 34, 80078 Pozzuoli, Italy; gandreotti@icb.cnr.it; 3 Stazione Zoologica “Anton Dohrn”, Villa Comunale, 80121 Naples, Italy

**Keywords:** lysosomal storage disease, lysosomal storage disorder, lysosomal enzyme disorder, drug repositioning, drug repurposing

## Abstract

Rare diseases, or orphan diseases, are defined as diseases affecting a small number of people compared to the general population. Among these, we find lysosomal storage disorders (LSDs), a cluster of rare metabolic diseases characterized by enzyme mutations causing abnormal glycolipid storage. Drug repositioning involves repurposing existing approved drugs for new therapeutic applications, offering advantages in cost, time savings, and a lower risk of failure. We present a comprehensive analysis of existing drugs, their repurposing potential, and their clinical implications in the context of LSDs, highlighting the necessity of mutation-specific approaches. Our review systematically explores the landscape of drug repositioning as a means to enhance LSDs therapies. The findings advocate for the strategic repositioning of drugs, accentuating its role in expediting the discovery of effective treatments. We conclude that drug repurposing represents a viable pathway for accelerating therapeutic discovery for LSDs, emphasizing the need for the careful evaluation of drug efficacy and toxicity in disease-specific contexts.

## 1. Introduction

Rare diseases, or orphan diseases, are defined as diseases affecting a small number of people compared to the general population. No standard definition exists for how “small” the number of patients should be, and different conventions are used worldwide. For example, the European Union considers “rare” a disease affecting fewer than five people in 10,000 (European Commission). In comparison, the Orphan Drug Act (United States) defines a “rare disease” as a disease or condition affecting less than 200,000 people (Orphan Drug Act, 1983) [1].

Databases and databanks dedicated to rare diseases, such as patient registries, are crucial for overcoming the research limitations posed by the small number of patients with each rare disease [2] and facilitate the description of the natural history and phenotypic diversity of rare diseases, improving case definition and indication to treat [3].

One of the most prominent resources in this field is OrphaNet, an initiative that began in France in 1997 and has since expanded to form a Europe-wide network [4]. OrphaNet offers an extensive database of rare diseases, providing detailed information on their prevalence, clinical signs, genetic causes, and available treatments Furthermore, OrphaNet organizes diseases in hierarchies [5], allowing for a systematic and structured classification of diseases and making it easier to navigate and understand the relationships between diseases and their subtypes.

In addition to OrphaNet, the Online Mendelian Inheritance in Man (OMIM) is another crucial resource. Managed by the Johns Hopkins University School of Medicine, OMIM is a comprehensive compendium of human genes and genetic phenotypes [6]. It focuses on the relationship between genotype and phenotype, offering detailed descriptions of genetic disorders, including their molecular basis and clinical features. This focus makes OMIM particularly useful for research into the genetic aspects of rare diseases.

Nearly all rare diseases (their number is estimated to be between 5000 and 8000, with 7000 representing the most commonly cited number [7]) are Mendelian [8]. Notwithstanding the considerable differences in their characteristics and phenotypes, they are all associated with diagnostic challenges, management, and the absence of cures.

Addressing the therapeutic needs of rare diseases presents a significant challenge due to factors like limited understanding, small patient populations, and high research and development costs [9]. These limitations also affect funding, which is a significant issue because rare diseases are not an attractive field for pharmaceutical companies, and public funding is limited [10,11,12]. Nevertheless, the latter typology is typically the primary source for research on rare diseases [11].

These obstacles often lead to a paucity of dedicated treatments, leaving many rare disease patients with few options. In this context, drug discovery is particularly challenging [13]. There are two main strategies: time-intensive de novo discovery, often not pursued by pharmaceutical companies due to low incentives (the aforementioned high costs and rarity of patients), and the increasingly prevalent drug repositioning. Drug repositioning involves repurposing approved drugs for new therapeutic applications [14], offering advantages in cost, time savings, and a lower risk of failure [15,16,17,18].

Lysosomal storage disorders (LSDs) are inherited rare diseases marked by lysosomal abnormalities resulting from the buildup of undegraded substrates. The buildup perturbates cellular balance and causes damage across various organ systems. Such dysfunction is typically due to mutation-driven deficits in certain lysosomal enzymes or crucial non-enzymatic proteins necessary for normal lysosomal operations.

As in the case of the vast majority of rare diseases [8], LSDs are generally inherited in an autosomal recessive manner: to develop the disorder, an individual needs to inherit two copies of the defective gene—one from each parent. However, some LSDs, like Fabry disease (FD), are inherited in an X-linked manner, which typically affects males more severely than females.

LSDs can be broadly classified based on the nature of the primary substance that accumulates due to the deficient enzyme (e.g., mucopolysaccharidosis and sphingolipidoses). Nonetheless, it is worth noting that each LSD has its own pathophysiology, clinical presentation, and genetic basis. Such diversity has implications for diagnosis, treatment, and management, necessitating tailored approaches for each disorder. Thus, treatment options for LSDs vary depending on the specific disorder and its severity. Some disorders can be addressed by providing patients with the enzyme they lack, using enzyme replacement therapy (ERT). Other treatments may include using small molecules (pharmacological chaperone therapy, or PCT) acting as chaperones to stabilize unstable yet functional mutants, substrate reduction therapy (SRT), and gene therapy.

PCT (alone and in conjunction with ERT) and SRT could benefit from drug repositioning. A representative example comes from glucosidase inhibitors, which were first investigated for the treatment of HIV-1 infection and then proposed as pharmacological chaperones (PCs) [19,20]. In such cases, it is crucial to underline that the efficacy of repurposed drugs needs to be tested with respect to each disease-causing mutation [21,22].

In this review, we directed our focus toward drug repositioning for LSDs. Our attention in this context is expressly paid to how drug repositioning has been leveraged to improve existing therapies for LSDs. Given the complexity and diversity of LSDs, traditional drug development faces numerous challenges, including the need for extensive time and resources to bring new treatments to the market. Drug repositioning offers a potentially more cost-effective alternative. Furthermore, it potentially permits bypassing some initial stages of drug development by exploring the use of already-approved drugs, which have well-characterized profiles regarding safety and pharmacokinetics. By delving into the potential of drug repositioning, we aim to provide a snapshot of the ongoing efforts to enhance therapeutic strategies for LSDs.

## 2. Drug Repositioning for Lysosomal Storage Disorders: Still a Niche Field

LSDs are inborn errors in metabolism caused by mutations in lysosomal enzymes. OrphaNet reports 143 nodes under the “lysosomal storage disease” root, with each node representing a disease or a disease family, while an OMIM semantic search for LSDs using the query ‘“lysosomal storage disease” OR “lysosomal storage disorder” OR “lysosomal disorder”’ generates 108 results, of which 36 are identified as genes and 72 as phenotypes at various degrees of understanding. The two lists were aligned through the Orphadata cross-referencing dataset [23] and manually enriched with gene and protein IDs. The result was saved as Supplementary Table S1 and was used as a reference when evaluating how representative our dataset is for LSDs diversity.

To evaluate the use of drug repositioning for LSDs, we performed literature mining on Scopus (accession date 12th December 2023) with the following query:

(“drug repositioning” OR “drug repurposing” OR “nutraceutical repositioning”) AND (“lysosomal storage disease” OR “lysosomal storage disorder” OR “lysosomal disease” OR “lysosomal disorder” OR “lysosomal enzyme disorder”).

We acknowledge that the selection of keywords, which is crucial for any data-mining endeavor, may have led to the omission of relevant studies. Therefore, it should be understood that our research was thorough within the confines of the methodology employed.

**Figure 1:**
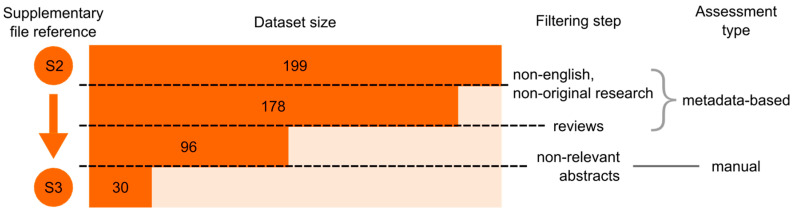
Visualization of the article selection process.

The research resulted in 199 articles (Supplementary Table S2), which underwent curation. Eliminating non-English-language entries, book chapters, surveys, editorials, retracted articles, and conference materials reduced the papers to 178. Among these, 82 reviews were excluded. The remaining 96 articles were manually screened based on an expert review of the available abstract to identify those of interest, and 30 were selected. Three articles were excluded since the full text was not available. Supplementary Table S3 contains the 27 articles included in the review. A workflow detailing the entire process is visible in Figure 1, and papers are synthesized in Table 1. Searching for these articles’ DOIs on UniProt returned two hits [24,25] linked to proteins not associated with any OMIM or OrphaNet on UniProtKB, while searching the publication DOIs on OMIM using the “ref_doi’’ key yielded no results. Furthermore, ~30% of the articles were not associated with any LSD-related Medical Subject Headings (MeSHs), and only five of those 27 documents were annotated on PubMed with the Drug Repositioning MeSH. The results of this cross-search are interesting as they identified the inclusion of the selected articles in searches based on domain identifiers (MeSH, OrphaNetID, OMIMID) as very unlikely.

Furthermore, the diversity of LSDs in the dataset was low: the highest number of papers belonged to FD (5) and the mucopolysaccharidosis subfamily (4). The total number of diseases considered (13) was a small subset (18%) of the LSDs population on the joint OMIM/OrphaNet list (Supplementary Table S1), even considering the most conservative count (OMIM phenotypes).

**Figure 2:**
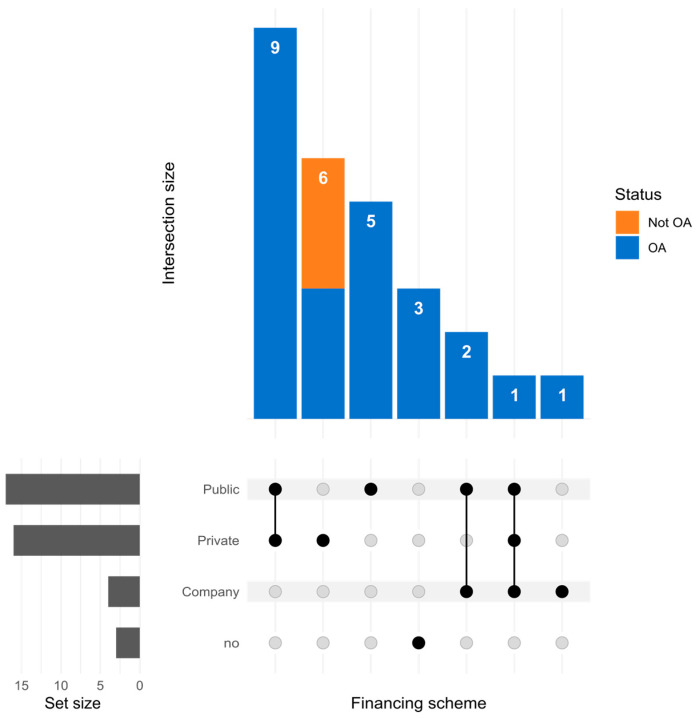
UpSet plot that conveys the funding distribution and access status of scholarly papers. Horizontal bars at the bottom visualize the total count of papers funded by each scheme. The vertical bars show the frequency of papers with multiple funding sources. Connected dots highlight the specific financing sources involved in each composition. The color coding distinguishes between open-access (OA, blue) and closed-access (Not OA, orange) papers, with the numbers atop the bars indicating the size of each intersection.

The funding source for each selected article was analyzed, and the results are reported in Figure 2. We classified the donors as public, private (including private universities or research institutes and foundations), or companies.

Almost all papers (24/27) received funding from some entity, mostly from joint efforts of the public and private sectors. For-profit companies represented a minority, and closed-access papers (three out of twenty-seven) were funded exclusively by the private sector. As Rubinstein et al. said [26]: “The value of Open Science is nowhere more important and appreciated than in the rare disease community”. Open Science, of which open access is a part, can significantly benefit the rare disease community by facilitating the sharing of data and knowledge. The minimal fraction of closed-access papers is a promising signal in this sense.

**Figure 3:**
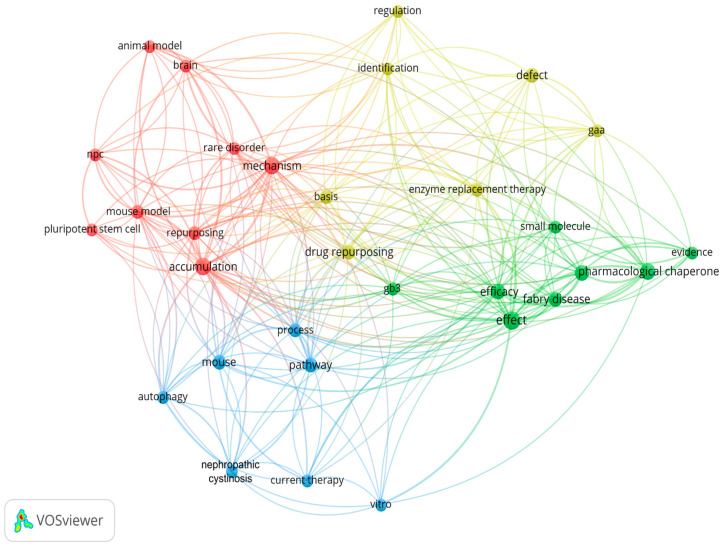
Term co-occurrence network of the 27 papers. Nodes are relevant terms present in at least three titles or abstracts; links represent contemporary presence. Colors mark groups of tightly knit terms (i.e., modularity clustering). Abbreviations: npc, Niemann–Pick disease; gaa, Pompe disease; gb3, Globotriaosylceramide.

The VosViewer^TM^ [27] semantic analysis of the 27 articles showed that the community is mainly dedicated to a few diseases, namely nephropathic cystinosis, Niemann–Pick type C (NPC), Pompe disease (PD), and FD (Figure 3).

## 3. Shooting at Random: The Valuable Results of High-Throughput Screening

The use of high-throughput screening (HTS)—i.e., broadly screening extensive compound libraries—represents a potent instrument for drug repositioning in rare diseases in general and LSDs in particular. Many successful repurposing processes started with HTS screening and were confirmed by targeted experiments. The desired effects usually fall within two categories: a direct effect on the deficient enzyme (i.e., using the drug as a PC) or an indirect effect. The latter can be obtained by substrate reduction, lysosomal activity regulation, or both. The following sections are arranged around this dichotomy. Each LSD will be briefly described upon its first mention to facilitate navigation through the text. Furthermore, due to the variety of diseases (13), we compiled a synthetic table listing genes, disease names, and symptoms for reference (Supplementary Table S4).

### 3.1. Repositioning HTS-Identified Drugs as Pharmacological Chaperones

Research efforts that repurpose HTS-identified drugs as PCs are less frequent compared to those investigating effects on lysosomal activity. This imbalance is likely due to the more challenging nature of hypothesizing a small molecule as a PC versus assessing an indirect effect (e.g., by analyzing transcriptomic data in differential expression studies).

Mucopolysaccharidosis type IV A (MPS IVA) is associated with mutations in the *GALNS* gene, and its clinical features are skeletal and joint abnormalities. Olarte-Avellaneda and co-workers proposed bromocriptine (BC) as a new PC to treat this disease [28]. Since its approval in 1978, BC therapeutic applications have been related to neurological disorders (in particular, symptomatic Parkinson’s disease and spastic and extrapyramidal disorders), hyperprolactinemia and acromegaly, and diabetes mellitus type II [29]. The authors started with an in silico screening of the ZINC In Man subset of the ZINC chemical compound database (In Man meaning experimental compounds that have been in man, worldwide, including drugs). Within this screening, they identified BC as a highly possible PC and tested this potential with a thorough characterization, including in vitro and ex vivo experiments.

Higaki et al. [30] showed the screening results for new chaperones to treat GM1-gangliosidosis, an LSD caused by a deficiency of the β-galactosidase enzyme [31]. Concurrently with the design of new chaperones for the enzyme, they described its response to approved molecules. In particular, they started from a library of chemical compounds approved for human administration and reported a table containing the mutation-specific behaviors. Among these molecules, the authors cited galactose, which is particularly interesting. They discussed the possibility of combining the use of PCs with ERT together with the ongoing trials focused on the use of 1-deoxygalactonojirimycin (DGJ) and 1-deoxynojirimycin, respectively, for FD and PD (see Supplementary Table S3).

Miura and co-workers [32] described another candidate PC for the same disease. Upon assessing a fluorogenic-based high-throughput screening for Golgi-associated β-galactosidase, they performed a compound screening, selecting an isoflavone derivative. At this point, they switched to rational drug design, optimizing the molecular structure of the potential drug by in silico docking. The obtained molecule, ARM07, represents a potent inhibitor directed explicitly to the Golgi-localized β-galactosidase. The authors discussed the possibility of selectively inhibiting this enzyme without altering the lysosomal form, thus paving the way to further understanding its different roles in cells.

### 3.2. Repositioning HTS-Identified Drugs as Regulators of Lysosomal Function

The other desirable effect sought in HTS for drug repositioning is the ability to modulate lysosomal function, e.g., through regulating the concentration or the availability of the substrate/product of the defective enzyme. In this sense, experiments using cellular and animal disease models to assess repositioning efficiency have proven crucial. This importance is evidenced by numerous studies using HTS to identify drugs that can regulate lysosomal function, primarily based on these models.

PD is a genetic disorder affecting muscle and nerve cells throughout the body, caused by an accumulation of glycogen due to a deficiency in the lysosomal enzyme acid α-glucosidase. The attention paid by Buratti and co-workers to a specific mutation common in late-onset PD patients led to the identification of deferoxamine (Defe) as a potential lead compound for improving the pathological phenotype [33]. The mutation of interest in the *GAA* gene was the intronic variant c.-32-13T>G, leading to the partial or complete removal of exon 2 in the mature mRNA [34,35,36,37,38,39]. The authors set up a GFP-based cell reporter system allowing the screening of drugs that improve exon 2 retention. The selection of candidates was carried out following an initial evaluation round that involved screening a library of 1280 drugs approved by the Food and Drug Administration (FDA). The second-phase tests led to the identification of Defe, whose effects were found to be mediated by iron availability. Notwithstanding the limitations of using such a drug in patients who do not present iron over-storage, it is of utmost importance for the Pompe community to have the possibility of developing modified molecules based on these results.

Nephropathic cystinosis is a form of cystinosis severely affecting the kidneys and other organs. Bellomo and co-workers combined a two-assay bench-based high-throughput screening with an in silico strategy to identify potential drugs for nephropathic cystinosis [40], a metabolic disease causing an accumulation of cystine within cells, leading to kidney problems and growth impairment. The results from a high-throughput screening based on cell cystine content and those from a high-content drug screening based on an apoptosis assay were then combined, and five drugs were identified that reduced the cystine content and prevented apoptosis (alexidine dihydrochloride, β-escin, digoxin, disulfiram (DSF), and fluspirilene). Alexidine hydrochloride is an anticancer agent, and it has been recently identified as an antifungal and antibiofilm agent [41,42]. β-escin and digoxin are plant-based drugs. The former is used for treating chronic venous insufficiency, based on its anti-inflammatory and anti-edematous properties [43]; the latter is approved for treating heart failure [44]. DSF is used for the treatment of chronic alcoholism [45], while fluspirilene is an antipsychotic drug [46]. The authors also performed a transcriptomic analysis to deepen the common pathways regulated by the identified drugs. They produced a list of additional potential drugs acting with the exact mechanisms of action. In a follow-up paper, the same authors investigated the effects of DSF on animal models using mice and zebrafish [25]. Notwithstanding the decades of DSF application, in particular, to treat chronic alcoholism, prolonged exposure to the drug resulted in markedly increased toxicity for cystinotic animals. As commented by the authors, these results shed light on the necessity of disease-specific drug evaluation, even in the case of drug repositioning.

Within HTS-guided repositioning experiments, lysosomal regulation has also been assessed in other ways. De Leo and co-workers identified impaired autophagic flux and an increase in the amount of SQSTM1 proteins (which are linked to autophagy) as nephropathic cystinosis markers [47]. They developed an in-cell ELISA screening process to identify drugs reducing the autophagy markers on this basis. The screening allowed them to identify six molecules of interest, and among these, they focused on luteolin. Luteolin is a flavonoid, and its properties have been exploited by Chinese traditional medicine for hypertension, inflammatory diseases, and cancer [48,49]. Besides repairing the autophagic defects in cystinotic cells, the authors demonstrated that luteolin protects from apoptosis and ameliorates oxidative stress, acting on different features of nephropathic cystinosis.

Batten diseases are a fatal, inherited group of childhood-onset disorders of the nervous system, leading to symptoms such as vision loss, seizures, and progressive motor and cognitive decline [50]. Soldati and co-workers presented the accumulation of globotriaosylceramide (Gb3) as a newly identified pathological mechanism in models for two members of the disease group (caused by mutations in the genes *CLN3* and *CLN7*) [51]. The *CLN3* models included a miniswine model with exon 7–8 deletion (*CLN3*Δex7/8) [52] that showed consistent and progressive Batten disease pathology and behavioral impairment. *CLN3* depletion in cells leads to the mis-trafficking of lysosomal enzymes and defective autophagic lysosomal reformation [53]. The *CLN7* models, on the other hand, included zebrafish individuals with mutations in the *CLN7* gene. These models exhibited higher basal activity, hyposensitivity to light changes, and hypersensitivity to pro-convulsive drugs. Phenotype observation led to the high-throughput screening of drugs specifically reducing Gb3, aiming at the improvement of the *CLN7*-associated phenotype. Over a panel of 1280 drugs, nine compounds were identified as worth exploring. The attention of the authors was focused on tamoxifen, an European Medicines Agency (EMA)- and FDA-approved drug that has been used for decades in the treatment of breast cancer and hormone-related disorders, as well as pediatric conditions [54]. The effect of tamoxifen in Batten disease is mediated by its action on transcription factor EB (TFEB), a master regulator of the lysosomal function in cells, which promotes the clearance of pathological storage in different LSDs [55,56,57,58].

Capuozzo and co-workers [59] also highlighted the central role of TFEB in treating mucopolysaccharidosis type IIIA (MPS IIIA), a disease associated with the accumulation of heparan sulfate and progressive neurodegenerative symptoms. The attempt to reposition drugs for MPS IIIA started with developing a high-content screening technology to evaluate the functionality of the cargo’s degradation by the lysosome based on self-quenched bovine serum albumin (DQ-BSA). FDA-approved molecule screening allowed the authors to identify fluoxetine as a drug of interest. Fluoxetine is among the most well-known antidepressants, acting on serotonin uptake [60]. In the discussed paper, serotonin was identified as improving lysosomal function by inducing autophagy and, most importantly, regulating TFEB. Translation to in vivo studies on MPS IIIA mice models demonstrated its efficacy in ameliorating the phenotype.

Multiple Sulfatase Deficiency (MSD) is a rare genetic disorder affecting multiple enzymes (e.g., *SUMF1*), leading to symptoms that include developmental delay and skeletal abnormalities. The screening of 785 FDA-approved drugs on MSD patient cells led Schlotawa and co-workers [61] to the identification of two agonists of retinoic acid receptors (RARs) and retinoid X receptors (RXRs)—respectively, tazarotene and bexarotene. These are retinoic acid derivatives, and their effects on restoring sulfatase activity are particularly relevant when used in combination with each other. The authors speculated that RAR/RXR heterodimers mediate sulfatase activation in MSD cells.

FD is caused by mutations in the *GLA* gene, and it affects the body’s ability to break down Gb3, leading to various symptoms, including kidney failure and heart issues. Iacobucci and co-workers characterized interactors for the available recombinant enzymes approved for ERT in FD (namely agalsidase α and agalsidase β) [62]. They mined the interactomes for entries known to interact with approved drugs, suggesting the investigation of these drugs for their effects on FD patients.

## 4. Meet Serendipity Halfway: Targeted Drug Repositioning

Another approach to drug repositioning involves targeting drugs that influence an established pathology’s phenotype or disease marker. This strategy is based on the rationale that the same marker/phenotype may also be relevant to LSDs, suggesting a shared pathological mechanism or symptom that can be addressed with the same therapeutic targets. For a concise description of the disease and a reference to the detailed description on OrphaNet/OMIM, please refer to Supplementary Table S3.

### 4.1. Symptoms as the Compass

Symptomatic evidence guided Bragato and co-workers to identify the potential of amifampridine (3,4-diaminopyridine phosphate, or 3,4-DAP) for treating PD [63]. 3,4-DAP increases acetylcholine concentrations at the neuromuscular junction and is used as a symptomatic treatment for myasthenic syndromes, which are characterized by neuromuscular junction impairment, a feature of PD [64]. Drug testing on a zebrafish model reduced neuromuscular junction impairment. The authors hypothesized that treatment could prevent such impairment, with higher effects in infantile-onset PD.

Recently, a hypothesis has been proposed for using D-cycloserine in treating Krabbe disease (KD) [65]. KD is a severe neurological condition that results from the deficiency of a specific enzyme, leading to symptoms like muscle weakness and intellectual decline, typically appearing in the first months of life. Preliminary experiments described by LeVine and Tsau provided evidence that treating twitcher mice with this antibiotic can slow the disease’s course. The authors discussed a putative mechanism of action based on data available in the literature, hypothesizing that the inhibition of serine palmitoyltransferase (SPT) is thus involved in substrate-reducing drugs. D-cycloserine, or seromycin, is a well-known broad-spectrum antibiotic mainly used in tuberculosis [66], but it has broad potential in treating neuropsychological disorders [67].

Given the emergent complexity of LSDs, defining drug repurposing efficacy is sometimes problematic. For example, ambroxol is a mucolytic drug mainly used for respiratory diseases [68] identified as a candidate PC for treating Gaucher disease (GD) [69], the most common LSD, characterized by a deficiency in lysosomal acid glucosylceramidase (*GBA1*), leading to organ damage, bone pain, and anemia. Nevertheless, its role in this LSD has never been clarified. In a paper by Pantoom and co-workers [70], the mechanism of action of ambroxol in GD was explored. Evidence provided by in vitro experiments led the authors to hypothesize the possibility that ambroxol does not act as a PC. Notwithstanding its efficacy, they suggested that the mechanism of action underlying its effects needs further investigation.

Sometimes, repositioning happens almost in real time with the case reporting of novel or exceedingly rare diseases. While reporting a case displaying mucopolysaccharidosis plus, a rare and attenuated subtype of mucopolysaccharidosis related to the VPS33A protein, Pavlova and co-workers described the effects of bortezomib and eliglustat in improving glycosphingolipid trafficking in patient-derived fibroblasts [71]. On the one hand, eliglustat is the drug approved for GD, and it acts as an inhibitor of glucosylceramide synthase, being used in substrate reduction therapies [72]. On the other hand, bortezomib is a proteostasis regulator that acts as an antineoplastic agent [73].

### 4.2. Mining Transcriptomics and Pathways

In cases of limited evidence and diseases with profound implications like LSDs, a data-driven approach could bring results suitable for exploitation by targeted repositioning. Identifying unique gene expression patterns, defining disease subtypes, and discovering relevant genes associated with unconsidered metabolic pathways disrupted in disease phenotypes can all aid in discovering potential therapeutic targets.

NPC, caused by mutations in the *NPC1* gene, involves the accumulation of lipids in the liver, spleen, and brain, leading to progressive neurological symptoms. Pepponi and co-workers, finding evidence that NPC mice show reduced extracellular adenosine levels [74], focused their attention on the possibility of increasing adenosine levels by inhibiting its equilibrative nucleoside transporter (ENT1) [75]. To this end, they used dipyridamole, an antiplatelet aggregation known to inhibit ENT1. Treatments were performed on NPC1-derived fibroblasts, and the drug’s efficacy was evaluated in terms of reduced cholesterol abnormal storage. Besides substrate clearance, the authors observed the rescue of mitochondrial membrane depolarization and increased extracellular adenosine.

Finally, Braun and co-workers established an FD model on podocytes using CRISPR/Cas9 technology [76]. Their results are of utmost importance in the field since they reported significant lysosomal dysfunction combined with podocyte injury upon ERT treatment, notably despite Gb3 reduction. Transcriptomic and proteomic analysis identified α-synuclein (*SNCA*) to be upregulated in the FD model and the only protein not restored at the physiological level upon ERT treatment. The investigation by the hyper- and hypo-expression of *SNCA* in wt and KO *GLA* cell lines confirmed its ability to recapitulate the FD phenotype when overexpressed. The use of β-adrenoreceptor agonists (clenbuterol and orciprenaline), known to decrease the risk of Parkinson’s disease by the down-regulation of *SNCA*, improved the FD phenotype in the KO *GLA* cells, particularly in the co-treatment of clenbuterol and ERT.

Along the same lines, Shimizu and co-workers focused on repurposing two FDA-approved drugs, minocycline and etanercept, for GD [24]. Using murine models, Shimizu et al. demonstrated that macrophage-inducible C-type lectin (MINCLE) activation is involved in the neuropathic symptomatology of GD. Combined treatment with minocycline, a microglial activation inhibitor, etanercept, and a tumor necrosis factor (TNF) blocker significantly improved the neurologic phenotype and prevented early death.

In a paper by Morales and co-workers, the effect of ursodeoxycholic acid (UDCA), a naturally occurring bile acid, was investigated in GM2-gangliosidosis [77], a disease marked by a harmful accumulation of certain fats in the brain and nerve cells, resulting in progressive neurological impairment. The authors reported the role of this molecule in reducing endoplasmic reticulum stress by directly binding and activating PERK (an endoplasmic reticulum kinase), promoting its auto-phosphorylation and stabilizing its dimerization. Furthermore, in their cell model of GM2-gangliosidosis, orally active UDCA reduced neurite atrophy.

NPC1 is a specific form of Niemann–Pick type C, leading to similar symptoms of fat accumulation and cellular dysfunction. Comparing single-cell RNAseq data, Cougnoux and co-workers identified a reduced expression of *Slc1a3*, which encodes a glutamate transporter, in NPC1 mice compared to wt [78]. Moving from this observation, they performed research to identify drugs explicitly acting on glutamate transport as potential treatments for the NPC1 disease. They demonstrated that riluzole improved the neurological phenotype in mice, mainly acting on astrogliosis, whose onset is late in the disease progression. This finding is coherent with what is currently known: riluzole is an amyotrophic lateral sclerosis drug acting as a neuroprotective inhibitor of presynaptic glutamate release [79].

Referring to FD, Delaleu and co-workers analyzed the transcriptomes from different kidney compartments before and after ERT and validated transcriptional landscapes associated with nephropathy [80]. Members of these pathways were analyzed to collect data about known drug–target interactions to identify potential drugs to be repurposed.

### 4.3. Using Drugs as a Template: Exploring Similarities in the Chemical Space

Oxysterols are intermediates in the cholesterol excretion pathway and play various physiological functions [81]. Based on the evidence that 25-hydrocholesterol (25HC) acts as a PC for NPC1, Ohgane and co-workers switched to rational drug design, producing many oxysterol derivatives and testing their chaperoning capability for NPC1 [82].

The use of proteostasis regulators in treating protein misfolding diseases is a hot topic in the literature [83]. Following this branch, Monticelli and co-workers described the potential of repurposing acetylsalicylic acid (ASA) to improve PCT for FD [84]. At first, they established a cell model starting from patient-derived immortalized fibroblasts with a large deletion of the *GLA* gene that encodes for the α-galactosidase protein (AGAL). These cells allowed the determination of the effects of drugs on different AGAL mutants with the same genetic background and the evaluation of the effects of long-term treatments. The combined treatment with ASA and DGJ (the approved PC for FD) revealed an increased intracellular AGAL stability, resulting in better substrate reduction upon long-term treatment. With the same approach, the authors explored the effect of a nutriceutical, curcumin. *Curcuma longa* L., a plant with a long history of usage in Chinese medicine, is the basis for this drug [85]. The growing interest in curcumin usage and the broad spectrum of its potential applications (ranging from cancer to different chronic and inflammatory diseases) recently led the Food and Drug Administration to recognize it as Generally Recognized As Safe (GRAS). Monticelli and co-workers highlighted the beneficial effect of curcumin in increasing AGAL in FD cell models, both alone and in combination with DGJ. Altogether, these results paved the way to re-modulate PCT, reducing the adverse effects of its administration.

## 5. Expanding Human Skills: In Silico Models

Coupling high-throughput data production with data infrastructure and computational powers makes it feasible to use machine learning (ML) to aid drug repositioning. ML methods could be implemented during the coupling of the known drug with the disease-causing protein by aiding the recognition of potential drug–disease associations (e.g., through information networks [86,87] or embeddings [88]). The same methods could produce valid drug combinations that potentiate the effect of the cure [89]. However, machine learning for drug repositioning needs to overcome two consistent challenges to achieve this. Firstly, the limited number of validated drug–disease associations makes it difficult for classification models to learn effective latent factors of drugs [90]. Additionally, existing models often rely on negative sampling techniques, which may not be valid in real-world settings [91]. According to our search, various computational methods have been applied to drug repurposing in LSDs.

The paper by Esmail and Danter is of utmost interest in this field [92]. Focusing on metachromatic leukodystrophy (MLD), a neurodegenerative disease affecting the metabolism of sphingolipids, they developed an in silico brain organoid model (aiWBO) based on the brain organoid platform NEUBOrg and machine learning platform DeepNEU. Their simulations produced a disease profile similar to that of the wet lab. Using such a model, they were able to observe new characteristics of the brain organoids. Besides this, they performed a high-throughput drug screening, leading to the identification of 42 top candidates. Interestingly, this group was principally made up of drug combinations, which were putatively put forward as the best treatment for MLD.

An entirely *in silico*, pathway-oriented approach that bridges different resources was described in the paper by Villalba and co-workers [93]. They started by assembling a list of differentially expressed genes from the Gene Expression Omnibus (GEO) transcriptomic data associated with mucopolysaccharidosis VII (MPS VII, related to glycosaminoglycan accumulation and multiple physical and developmental issues) and then performed enrichment analysis to identify relevant metabolic pathways. They cross-referenced the results with drug–pathway association databases like LINKS and CMAP and used the drugs found through this screening as targets for molecular docking. The pipeline identified two drugs (pirfenidone and colchicine) as promising repositionable treatments for cardiovascular issues in MPS VII patients.

An exciting database was described in the paper by Abdelhakim and co-workers, who developed the Drug Database for Inborn Errors of Metabolism (DDIEM) [94]. The DDIEM collects data available in the literature about treatments for Inborn Errors of Metabolism, integrating different types of information, from case reports to clinical trials. As the authors state, such a complete database represents an essential tool in drug repurposing.

## 6. Conclusions

Drug repositioning, or repurposing, is a strategy to identify new therapeutic uses for existing drugs [95,96,97], a promising approach with several limitations. Repositioned drugs are not so immediately applicable to therapy. The bench-to-bedside journey is shortened, but dosage and timing optimization for different diseases are required. Moreover, rare diseases are often infantile, and the repurposed drugs may not have appropriate dosing schemes for this specific group. Hence, repositioning often requires drugs to undergo phase II and III clinical trials for their new purpose, which can be time-consuming and costly [3]. Furthermore, large pharmaceutical companies’ business models and patent issues can hinder drug repositioning efforts [4].

The large number of rare diseases, each associated with a few patients, complicates drug discovery, requiring considerable efforts and cooperation among the members of different communities. In this context, repurposing approved drugs is fundamental, minimizing costs and timing and helping patients and their families to keep hope alive.

Historically, after examining a pathological phenotype that reveals aberrant protein expression, the primary investigation strategy involved identifying a drug capable of correcting this anomaly. A comprehensive literature review was then conducted to identify compounds previously used to address similar situations, aiming at repurposing those compounds. HTS and in silico techniques expanded this framework and permit an increase in the discovery rate.

In this review, we briefly discussed the strategies undertaken by researchers to profit from drug repositioning in the field of LSDs. We saw how the screening can be informed by the symptoms, chemical similarity of compounds, and knowledge of metabolic processes underlying the disease. HTS and differential expression analyses have been widely used in the papers we analyzed to explore the drug space and characterize targets. PCT also emerged as offering promising prospects for the treatment of LSDs. Many challenges, including safety and mutation-dependent suitability, must be overcome to make PCT effective and accessible to all patients affected by these diseases. Coupling drug repositioning with HTS techniques, expert knowledge, and in silico methods could reduce safety risks and facilitate mutation-specific evaluation.

## Figures and Tables

**Table 1:** AI-assisted synthesis of the 27 papers considered in the manuscript.

DOI	Synthesis
10.3390/ijms23073536	Ambroxol showed limited in vitro chaperone capabilities for GD, requiring further investigation into its mechanism of action.
10.3390/cells10123294	DSF demonstrated cystine-depleting and anti-apoptotic effects in nephropathic cystinosis but has dose-dependent toxicity and fails to prevent renal lesions.
10.3389/fped.2021.807973	D-cycloserine extended the lifespan of twitcher mice, suggesting potential as a therapy for KD through partial SPT inhibition and other therapeutic mechanisms.
10.1016/j.biopha.2021.111357	3,4-DAP showed potential for treating PD-related neuromuscular transmission dysfunction, improving neuromuscular junction structure and zebrafish behavior after treatment.
10.3390/ijms24087209	UDCA reduced endoplasmic reticulum stress-induced neurite atrophy and pro-apoptotic signaling in GM2-gangliosidosis models, suggesting a therapeutic role.
10.1016/j.immuni.2023.01.008	Minocycline and etanercept protected neurons and ameliorated symptoms in a GD model by limiting microglia activation and TNF-induced neuron phagocytosis.
10.3390/ijms24054548	Combining enzyme replacement with pharmacological chaperones or targeting AGAL interactors offers the potential for optimizing FD therapy.
10.1016/j.ymgme.2021.11.008	Decreased *SLC1A3* expression in NPC1 astrocytes suggests that glutamate uptake impairment contributes to disease progression; riluzole treatment improves survival.
10.3390/life12122085	Through repositioning analysis, pirfenidone and colchicine were identified as promising drugs for treating cardiovascular disease in mucopolysaccharidoses.
10.3390/ijms23073456	Increasing adenosine levels with dipyridamole reduced cholesterol accumulation and mitochondrial deficits in NPC, offering a new therapeutic approach.
10.1002/humu.24479	A study revealed a novel *VPS33A* mutation causing a rare mucopolysaccharidosis-plus-related variant, characterized by impaired intracellular trafficking, and suggested potential treatments using bortezomib and eliglustat to improve cellular functions.
10.15252/emmm.202013742	Research on Batten diseases identified tamoxifen as a potential treatment, showing it reduced harmful accumulation in cellular models and improved symptoms in a mouse model, highlighting the drug’s therapeutic promise.
10.1016/j.kint.2023.06.029	An analysis of Fabry nephropathy suggested that ERT can significantly reverse gene expression patterns in patients, with identified drug targets for potential treatment enhancement.
10.1016/j.omtm.2020.11.011	A screening for PD identified Defe as a drug that can restore normal gene splicing, offering a new therapeutic approach for patients with suboptimal response to current treatments.
10.15252/emmm.202114837	In MSD, tazarotene and bexarotene showed potential for correcting pathophysiology, marking a step towards developing treatments for this condition.
10.1016/j.ymthe.2022.01.037	Fluoxetine was repurposed for treating MPS IIIA, showing promise in reducing disease pathology in mouse models and suggesting a new treatment pathway through increased lysosomal exocytosis.
10.3390/ijms23095105	Research identified ASA as a potential enhancer for FD PCT, indicating a synergistic approach to treatment.
10.1172/JCI157782	A study revealed that ERT reduces globotriaosylceramide in FD but does not reverse lysosomal dysfunction, suggesting SNCA as a new therapeutic target.
10.3390/ijms24021095	Curcumin showed potential as a co-chaperone in treating FD, enhancing enzyme activity and lysosomal function in a cell model and advocating for personalized therapies.
10.3390/ijms222312829	A drug repurposing strategy for nephropathic cystinosis identified potential treatments, highlighting the approach’s effectiveness in addressing the therapeutic challenges of rare diseases.
10.3390/biomedicines9040440	The study utilized artificial brain organoids to explore MLD pathogenesis, offering new insights into potential therapeutic options through computer simulations.
10.1248/CPB.C20-00194	A novel Golgi β-galactosidase inhibitor, ARM07, showed potential in addressing LSDs and aging cell biomarkers, promising advancements in therapeutic probe development.
10.1681/ASN.2019090956	Luteolin emerged as a promising treatment for nephropathic cystinosis, enhancing autophagy-lysosome pathways and exhibiting antioxidant and anti-apoptotic properties, suggesting a potential breakthrough in renal LSD therapy.
10.1016/j.bmcl.2014.05.064	Oxysterol derivatives corrected folding defects in NPC, suggesting their utility as pharmacological chaperones for mutant NPC1 proteins, indicating a promising therapeutic strategy.
10.1186/s13023-020-01428-2	The DDIEM compiled therapeutic strategies for metabolic diseases, offering a new ontology-based knowledge base to guide treatment and management.
10.1021/acsmedchemlett.0c00042	BC was identified as a novel pharmacological chaperone for MPS IVA, showing the potential to increase enzyme activity and reduce lysosomal mass, highlighting a new therapeutic avenue.
10.4155/fmc.13.123	Chemical chaperones offer promising treatments for lysosomal storage and neurodegenerative diseases by stabilizing mutant proteins, emphasizing the potential for oral administration and blood–brain barrier penetration.

## Data Availability

Not applicable.

## Supplementary Materials

The following supporting information can be downloaded at: https://www.mdpi.com/article/10.3390/genes15030290/s1. Table S1, OMIM and Orphanet cross-referenced LSDs information. Table S2, Results of literature mining in Scopus. Table S3, Selected articles for the review. Table S4, Synthetic table listing genes, disease names, and symptoms of diseases analysed in the review.

## Abbreviations

**3,4-DAP** amifampridine or 3,4-diaminopyridine phosphate; **AGAL** α-galactosidase; **ASA** Acetylsalicylic acid; **BC** Bromocriptine; **DDIEM** Drug Database for Inborn Errors of Metabolism; **DGJ** 1-deoxygalactonojirimycin; **DSF** disulfiram; **Defe** Deferoxamine; **EMA** European Medicines Agency; **ENT1** equilibrative nucleoside transporter; **ERT** enzyme replacement therapy; **FD** Fabry disease; **FDA** Food and Drug Administration (US); **GALNS** galactosamine (N-acetyl)-6-sulfatase; **GBA1** lysosomal acid glucosylceramidase; **GD** Gaucher disease; **GEO** gene expression omnibus; **GRAS** generally recognized as safe; **Gb3** Globotriaosylceramide; **HTS** high-throughput screening; **KD** Grabbe disease; **LSD** lysosomal storage disorder; **MINCLE** macrophage-inducible C-type lectin; **ML** machine learning; **MLD** metachromatic leukodystrophy; **MPS IIIA** mucopolysaccharidosis type IIIA; **MPS VII** mucopolysaccharidosis type VII; **MSD** Multiple Sulfatase Deficiency; **PC** pharmacological chaperone; **PCT** pharmacological chaperone therapy; **PD** Pompe disease; **PERK** protein kinase R (PKR)-like endoplasmic reticulum kinase; **RAR** retinoic acid receptor; **RXR** retinoid X receptor; **SNCA** α-synuclein; **SPT** serine palmitoyltransferase; **SQSTM1** Sequestosome-1; **SRT** substrate reduction therapy; **SUMF1** Formylglycine-generating enzyme; **TFEB** transcription factor EB; **TNF** tumor necrosis factor; **UDCA** Ursodeoxycholic acid.
